# Suit-type Wearable Robot Powered by Shape-memory-alloy-based Fabric Muscle

**DOI:** 10.1038/s41598-019-45722-x

**Published:** 2019-06-24

**Authors:** Seong Jun Park, Cheol Hoon Park

**Affiliations:** Department of Robotics & Mechatronics, Korea Institute of Machinery & Materials, Daejeon, 34103 Korea

**Keywords:** Mechanical engineering, Electrical and electronic engineering

## Abstract

A suit-type wearable robot (STWR) is a new type of soft wearable robot (SWR) that can be worn easily anywhere and anytime to assist the muscular strength of wearers because it can be worn like normal clothes and is comfortable to wear even with no power supply. This paper proposes an STWR, in which a shape-memory-alloy-based fabric muscle (SFM) is used as the actuator. The STWR, which weighs less than 1 kg, has a simple structure, with the following components: SFMs, wire encoders for measuring the contraction length of the SFMs, and BOA that fix the actuators on the forearms. In this study, a position controller for the SFM using the wire encoder was developed, and a prototype STWR was fabricated using this position controller. Moreover, by putting the STWR on a mannequin, step-response experiments were performed in which the arms of the mannequin lifted barbells weighing 2 kg and 4 kg to a certain target position. A fast response of moving to the target position in less than 1 s was observed in all steps except for the initial heating step for the 2 kg barbell. The response speed of the SFM was noticeably slower for the 4 kg barbell compared to that for the 2 kg barbell; it moved to the target position in approximately 3 s in all the steps except for the initial heating step. The SFM-applied STWR could overcome the limitations of conventional robots in terms of weight and inconvenience, thereby demonstrating the application potential of STWRs.

## Introduction

Wearable robots constitute a technology that enhances and assists the physical functions of human arms or legs to exhibit a large force by using a small force. By enhancing the muscular strength of elderly persons or patients facing difficulties during movements in daily life, these robots help them move; in industrial sites, they can increase productivity by assisting workers to carry or move heavy objects. This technology of wearable robots is being researched and developed for several diverse applications. At present, it is one of the most spotlighted technologies^1–4^. Wearable robots are classified into hard-type wearable robot (HWRs) and soft-type wearable robot (SWRs) according to the materials used in the system and the maneuvering method. In an HWR, the whole system consists of a hard frame, and it is driven by mainly a motor-gear mechanism^3^. Because it delivers a force that is generated by the actuator to the human body through the hard frame, the assistive force can be easily delivered to the wearer. However, because the total weight of system is high and it is bulky, an HWR is inconvenient to wear. Furthermore, when no power is supplied, the range of activity is limited because the wearer cannot perform activities freely and the HWR becomes a burden. For example, the PERCRO body extender developed by Fontana *et al*.^5^ uses a direct-current brushed torque motor for the actuator and consists of an aluminum frame, and the total weight of system is 160 kg. In the NESM developed by Simona *et al*.^6^, the total weight of system is 136 kg, and the weight of the wearable exoskeleton module only is 13 kg. Furthermore, the exoskeleton developed by Innophys^7^ uses McKibben-type pneumatic artificial muscles for the actuators, and because it is driven by a manual pump, the total weight is 5.0 kg. Although its weight is relatively light, it is still bulky and inconvenient for wearers. To overcome the limitations such as the heavy weight and discomfort associated with HWRs, studies on SWRs have been actively carried out in recent years^8,9^. As an SWR consists of flexible materials, rather than hard materials, the weight is light and a person can wear it easily. Moreover, it is comfortable to wear, without any unfamiliar sensation. Most SWRs developed till date have motor^9–13^ or pneumatic-type^14–25^ actuators. A soft exosuit developed at Harvard Univ^10,11^. delivers force to a wearer by using a tendon-driven mechanism in which a motor winds and unwinds a wire. Although it has the advantage of a fast motor response, it also has a disadvantage in that a wearer has to additionally carry the actuator of the tendon-driven mechanism. The exosuit developed by Wehner *et al*.^14^, the SWR developed by Park *et al*.^15^ and the soft pneumatic glove developed by Polygerinos *et al*.^16^ use pneumatic actuators. Such pneumatic SWRs provide the advantages of fast response speed. However, they require devices such as compressors and regulators in addition to the actuator controller. Hence, actuating noise is high and the total volume and weight increase. Various soft actuators have been studied to overcome these problems. Textile/fabric-type actuators have been investigated, such as a fabric actuator that uses pneumatic tubes by Funabora^21^ and pneumatic textile actuator (PTA) by Fujimoto *et al*.^23^. In addition, actuators driven by electric voltage or electric Joule heating have been examined^26–31^. Maziz *et al*. studied soft actuator fabricated in textile form by knitting fibers with conductor coating^26^. However, its force was as small as 1 mN and its actuation time for one cycle was as slow as 800 s. Han *et al*.^28^ evaluated a textile/fabric-type actuator by wrapping a shape memory alloy with fiber and then knitting it like a yarn to realize a flower that spreads its petals at a temperature of approximately 70 °C by passing electric current or changing external temperature. This textile actuator is not suitable as an SWR actuator because of its weak strength and slow response speed. Yuen *et al*.^29^ developed a fabric actuator capable of bending motion by integrating a shape memory alloy wire on the fabric. Its strain under the unconstrained condition is as high as 60%, but the linear isometric force is only 9.6 N, which is insufficient for the SWRs. Ramachandran *et al*.^30^ developed an all-fabric wearable electroadhesive clutch capable of sustaining a 10 kg load, but this is not an actuator. As shown above, the textile/fabric actuators developed until now provide the advantages of flexibility and light weight. However, they are not suitable to be used as SWR actuators because of low load capacity and low strain.

**Figure 1:**
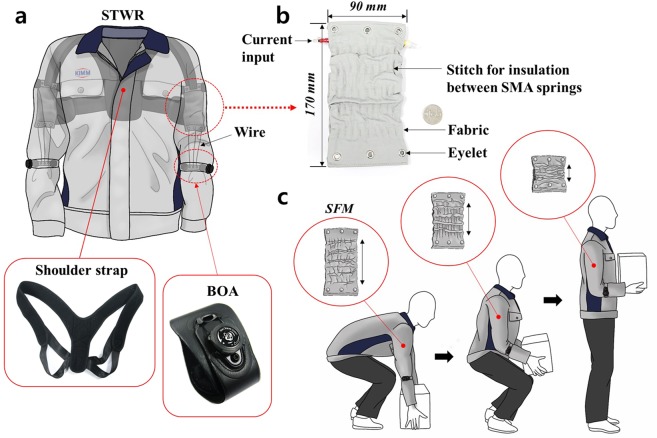
Overview of the SFM-applied STWR: (**a**) Schematic of the STWR. Inside the STWR, a shoulder strap is stitched and fixed to the back part of the garment; it connects the SFM and the body of the wearer. The SFM is placed between the shoulder strap and BOA^36^ attached to a sleeve; in reality, the STWR is worn while the BOA is in a loose state, and when the wear requires assistive force from the STWR, the BOA is fixed on the forearm. (**b**) Fabricated SFM. For insulation between the SMA springs inside the SFM, it is stitched between the SMA springs. The SFM consists of 20 SMA springs, arranged as two layers with each having 10 springs. (**c**) When a person wearing the STWR lifts an object and holds it, the relaxed SFM inside the STWR contracts and assists the muscular strength of the arms.

To expand the application range of SWRs, the limitations of conventional SWRs should be overcome. In other words, a silent SWR should be developed that is inexpensive, light, and easy to wear like normal clothes and can be worn comfortably even when there is no power supply. Therefore, in this study, a new suit-type wearable robot (STWR) was developed, which can provide assistive force to the arms of wearers, as shown in Fig. 1(a); this STWR comprises a shape-memory-alloy (SMA)-based fabric muscle (SFM) (Fig. 1(b)), which is an actuator that is inexpensive, light, flexible, and soft. An SFM is a fabric-type soft actuator composed of SMA spring bundles^31^. SMA coil springs fabricated from a NiTi SMA wire (NEXMETAL Co.) with a diameter of 0.5 mm and a transition temperature of 40 °C^32^ are arranged inside an SFM. Because the exterior is covered by a fabric, it is flexible, light, and soft. Table 1 compares the SFM used in this study with the earlier textile/fabric actuators. Pneumatic-driven PTA^23^ is 10 times heavier than the SFM. Electric voltage^26^ or electric Joule heating actuators^28,29^ are lighter than the SFM; however, their force is less than 1/10 of that of the SFM and they have lower strain. The SFM has a suitable force and strain performance for soft wearable robots compared to other textile/fabric-type actuators. Because the SFM is covered by a fabric, even if SFM directly touches the body of wearers, there is no discomfort. Furthermore, unlike other STWRs, because the SFM-applied STWR does not need additional devices such as motors, compressors, and regulators, it facilitates free movements and activities that have no limitation in terms of the movement range. Furthermore, because the STWR weighs less than 1 kg and is light as normal clothes, its wearability is excellent and daily activities can be performed anywhere and anytime. Moreover, it can be easily worn or removed without the help of another person.

**Table 1:** Comparison of textile/fabric-based actuators.

Actuation method	Fujimoto *et al*.^23^	Maziz *et al*.^26^	Han *et al*.^28^	Yuen *et al*.^29^	Park *et al*.^31^ (SFM)
	Pneumatic silicon tube	Conducting polymer (Electric voltage)	Shape memory alloy (Electric Joule heating)	Shape memory alloy wire (Electric Joule heating)	Shape memory alloy (Electric Joule heating)
Isometric force [N]	100	0.1	10.5	9.6	120
Strain [%] (Unconstrained condition)	18	0.32	4	60	67
90% Contraction Time [s]	—	620	25	31	1
Weight [g]	300	—	—	—	24

The SFM applied to the proposed STWR was designed and fabricated as described in^31^. 20 SMA springs which were fabricated using SMA wires of diameter 0.5 mm were used in SFM. According to the connection method used for the SMA springs inside the SMA, the total combined resistance changes; based on this change, the electric power required for driving the SFM is determined. To operate the SFM with a regular battery of capacity 15 V and 10 A, this study used an SFM fabricated by connecting four sets of SMA spring bundles in series, in which five SMA springs are connected in parallel^31^. The SFM, which contracts in the length direction owing to Joule heating, shows a 52% contraction in no-load conditions and 40% contraction under a load of 5 kg when heated from 25 °C (average room temperature) to 40 °C. Furthermore, because a single SMA spring can exert a force of 5 N with a 7.5 W input power, one SFM unit can exert a force of 100 N with an input power of 150 W. The force generated by the SFM is proportional to the number of SMA springs present in it^31,33,34^. The assistive force that can be exerted by the STWR is determined by the force specifications of the applied SFM. Therefore, according to the assistive force specifications of the STWR to be designed, the number of SFMs or the number of SMA springs in an SFM should be selected.

As shown in Fig. 1(c), when a wearer lifts a heavy object and moves while holding it, the SFMs attached to the STWR contract and assist the muscular strength of the arms. When a wearer performs a certain task, the contraction length of the SFM should be adjusted according to the circumstance. To this end, position control is required, which controls the length of the SFM by controlling the input current supplied to the SFM by measuring and feeding back the length of the SFM. To measure the length of the SFM, a small wire encoder that contracts and relaxes in the length direction was fabricated and attached to the SFM. This paper discusses the experimental results regarding the position control performance of SFM using the wire encoder, and the process for fabricating the STWR based on these results. Further, a mannequin was made to wear the STWR to evaluate its feasibility by performing force assistance performance tests and the arm angle control tests.

### Position control of SFM with a wire encoder

**Figure 2:**
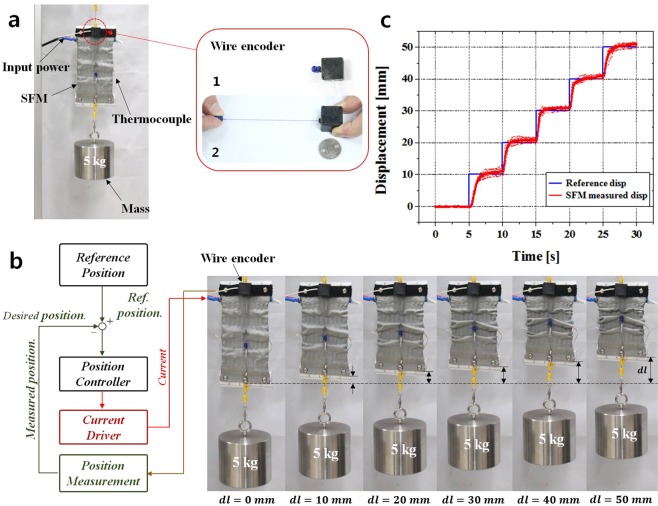
(**a**) Experimental setup for evaluating position control performance of SFM: (a1) Initial state of wire encoder. (a2) Wire encoder during pulling of the wire. (**b**) Block diagram of position controller for SFM. The SFM stretched by the 5 kg mass is controlled from the initial position (dl = 0) to a target position in steps of 10 mm. (**c**) Step responses of SFM.

To support the tasks of wearers, STWRs should be controllable according to the angle of the elbow, which changes depending on the task-performing states of wearers. Therefore, among the functions of the actuator that drives the STWR, position control is a function that is absolutely required. Hence, for the position control of SFMs, a wire encoder (Fig. 2(a2)) was fabricated and attached to the upper part of the SFM. The wire encoder (Custom-built by i2A systems Co., Ltd, Republic of Korea) is a sensor that measures linear displacement using a magnetic encoder (Fig. 2(a1))^35^. As shown in Fig. 2(a2), when the wire of the encoder is pulled, a voltage corresponding to the pulled length is outputted. Considering the contraction displacement of the SFM, the encoder was designed to measure displacements in the range of 0–100 mm; its width and length are 22 mm, height is 17.5 mm, and weight is 10 g.

Figure 2(a) shows the experimental setup for position control of the SFM after performing calibration to convert the voltage value outputted by the wire encoder into length units. The upper part of the SFM was fixed on the frame, and a 5 kg mass was hung at the lower part. The body of the wire encoder was attached to the upper part of the SFM, and the tip of the wire was fixed on the lower part of SFM. The displacement of SFM was measured by the wire encoder. In addition, a thermocouple for monitoring the temperature of the SFM was attached one of the SMA springs inside the SFM.

A proportional-integral (PI) position controller (Fig. 2(b)) was constructed for measuring the displacement of the SFM through the wire encoder attached to the SFM; by feeding it back, the input current supplied to the SFM was controlled to maintain the target position. A position control experiment was performed, in which the SFM was contracted into a staircase shape by increasing the target position in 10-mm intervals in the 0–50 mm range. Here, the displacement and temperature of the contracting SFM were measured simultaneously.

By adjusting the gain values of the PI position controller as K_p_ = 0.8 and K_i_ = 0.1, optimal gain values were set. In the remaining sections excluding the 0–10 mm section where the SFM contracted for the first time, a fast response was observed as the contraction to the target position was performed in less than 0.5 s. The 0–10 mm step showed a relatively slower response compared to the other steps because it is the step where the SFM is heated from an initial temperature of 25 °C. During position control of the SFM in the range of 0–50 mm, the measured maximum temperature was approximately 45 °C. For a contraction displacement of 50 mm, the temperature had to be increased by approximately 20 °C from 25 °C to drive the SFM.

### Fabrication and evaluation of the STWR based on SFM

**Figure 3:**
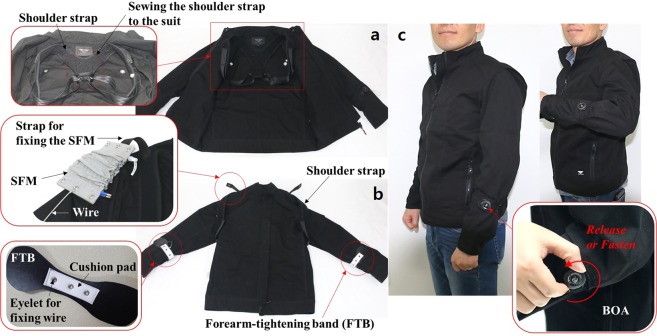
Fabricated STWR: (**a**) Interior of the STWR. On the back part, the shoulder strap is sewn and fixed. (**b**) The STWR is turned inside out to show the configuration inside the STWR. By attaching additional straps to the fixed shoulder strap, the SFM is fixed. An FTB on the lower sleeve is connected to the SFM via wires. (**c**) A human wearing the STWR. The dial of the BOA located on the forearm is turned to fasten or release the FTB on lower sleeve. Even with the FTB fixed, the arm-bending motion is comfortable and activities can be freely performed.

The STWR designed using the SFM and wire encoder described above was fixed to work clothes that can be easily purchased. The shoulder strap attached inside the STWR was fixed by sewing it on the back part of the garment (Fig. 3(a)). Moreover, to place the SFM around the biceps, a 100-mm-long strap was additionally sewn and attached to the shoulder strap (Fig. 3(b)). To replace the SFM easily, the upper part of the SFM was fixed by using the eyelet at the tip of the attached strap. Furthermore, the shoulder strap is fitted tightly on the shoulder and back of the wearer inside the STWR to prevent slipping or inclination of the SFM to one side caused by the load. Forearm-tightening bands (FTBs) were fabricated and attached to the lower sleeves of the garment to fix them to the lower part of the SFM using wires. Because the FTB is a part that contacts the forearm of wearer directly and is fixed, a cushion pad fabricated with a soft spongy material is attached to the internal contact surface (Fig. 3(b)).

For selectively fastening and releasing the FTB, a BOA^36^, which is often used in hiking shoes, running shoes, and medical assistive devices was used. Because the BOA is fastened when the dial connected to the wire is turned and the connected wire is wound, the FTB can be easily fixed on the arm by adjusting it to the thickness of the arm. During normal activities, if the FTB is released by releasing the BOA, it is in the same state as the sleeves of regular clothes; there are no restrictions for the activities and no hindrances for movements. When the wearer needs reinforcement or assistance for muscular strength, the FTBs are fixed on the arms by fastening the BOA (Fig. 3(c)). The total weight of the STWR is 0.96 kg, including all components such as the shoulder strap, SFM, and sensor, and it is similar to the weight of a regular jumper.

**Figure 4:**
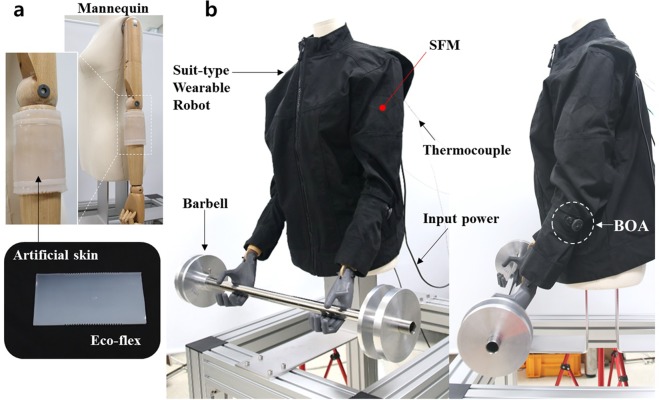
(**a**) Artificial skin made with silicone attached to the wooden arms of the mannequin to imitate human skin. (**b**) Experimental setup for evaluating position control performance of STWR. The mannequin wearing the STWR is holding up a barbell with both arms.

To evaluate the performance of the fabricated STWR, the experimental setup was configured as shown in Fig. 4(b). After putting the STWR on the upper body of mannequin, the arm-bending motion of the mannequin and position control performance of the STWR was evaluated. For this, two different loads were applied to the arms of the mannequin using barbells of weight 2 kg and 4 kg. By fastening the BOA, the FTB was fixed to attach the STWR on the lower forearms of the mannequin. As the mannequin had wooden arms, which are hard, unlike the soft arms of humans, the FTB could not be properly fixed. Therefore, an artificial skin was fabricated with silicone (Ecoflex 00–30, Smooth-On, Inc, USA) and attached to the mannequin to imitate human skin (Fig. 4(a)). The step-response performance was evaluated where the position was controlled according to a target displacement using method similar to that presented in Fig. 2(b) using the wire encoders of the SFMs attached inside the STWR.

**Figure 5:**
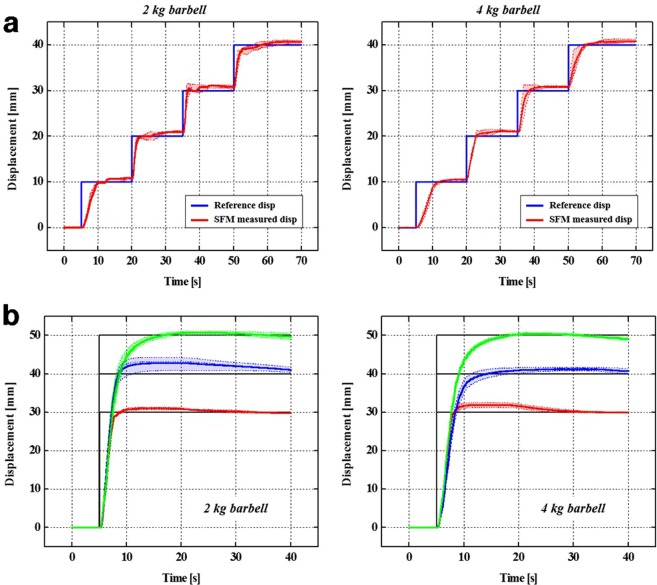
(**a**) Step responses measured when the mannequin wearing the STWR held barbells of weights 2 kg and 4 kg with its arms. (**b**) Responses when the target position was changed from the initial position of 0 mm to target positions 30, 40, and 50 mm, and then maintained.

**Figure 6:**
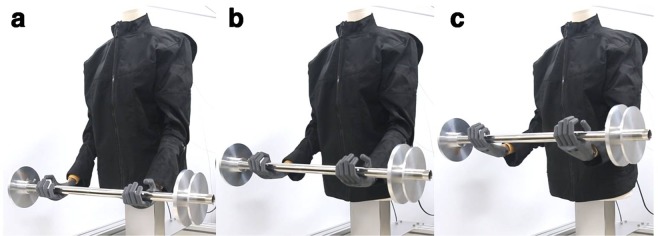
Contraction of the SFM inside the STWR causing the arms of the mannequin to lift the 4 kg barbell and move to a target position: (**a**) Initial arm position. (**b**) Arm position during 20-mm contraction of SFM. (**c**) Arm position during 40-mm contraction of SFM.

As shown in Fig. 5(a), when lifting the 2 kg barbell, a fast response of moving to the target position in less than 1 s is observed in all the steps excluding the 0–10 mm step, which was the initial heating step. Furthermore, when the weight of barbell was increased to 4 kg, the response speed of the SFM was noticeably slower compared to that for the 2 kg barbell; it moved to the target position in approximately 3 s in all the steps other than the 0–10 mm step. The larger the weight, the slower is the moving speed; however, we confirmed that for the SFM-applied STWR, position control could be performed in 10 mm intervals under loads, and using the generated assistive force, the arms of the mannequin could lift the 4 kg barbell (Fig. 6).

Figure 5(b) shows the step-response results in lifting the 2 kg and 4 kg barbells when moving from the initial position of 0 mm to target positions 30, 40, and 50 mm. Less time was consumed to reach the target positions when lifting the 2 kg compared to that when lifting the 4 kg one. Here, the SFM operated at a temperature of 45 °C or less when moving to target positions of 30 and 40 mm. However, when moving to a target position 50 mm, the temperature of the SFM rose to the maximum value of 70 °C. To reach a higher target position, 50 mm, the temperature of the SFM increased momentarily because of a high input current applied momentarily. After the SFM reached the target positions, the temperature was saturated at 37, 45, and 60 °C at 30, 40, and 50 mm, respectively.

When lifting the 2 kg barbell, the assistive force was generated at a level corresponding to the speed at which human arms lift an object; however, the speed was slower for the 4 kg barbell. Therefore, to produce assistive force at a faster rate for a heavy load, an SFM with a higher force capacity should be used. If the Joule heating rate is accelerated by increasing the input power to the SFM, the contraction speed of the SFM can be increased. However, because the natural cooling rate of a heated SMA spring is slow, the relaxation time required for the SFM is approximately 30 s. Therefore, the fabricated prototype STWR is difficult to apply in the tasks requiring bending and straightening of the arms. Nevertheless, it will be helpful for delivery workers who have to lift and hold heavy objects, as shown in Fig. 1(c).

## Discussion and Conclusion

This study developed a process of fabricating an STWR using SFMs and wire encoders. The position control performance the STWR under different load conditions was evaluated. Based on these evaluation results, the feasibility of the STWR was investigated; the STWR helps the arms of wearers to move to a desired position and exerts an assistive force. The STWR has a simple structure in which the SFM, wire encoder, and BOA are attached inside regular clothes, and it is easy to wear. Furthermore, because the internal actuators and fixed parts are all composed of fabric materials, the STWR is soft and flexible. Particularly, the STWR can be produced in various shapes. For example, using only the shoulder strap of Fig. 1(a), it can be fabricated in the form of a vest, which can be worn directly over outerwear by a worker, elderly person, or physically challenged person. Furthermore, by modifying the STWR such that it can assist other human body parts such as the shoulders, neck, legs, and waist, the muscular strength of wearers can be assisted in diverse work environments. Because the SMA springs have to retain their heated state to maintain the contracted state of the SFM, the electric current has to be supplied continuously, and this reduces the running time of the battery. To reduce power consumption, a mechanism should be developed to maintain the contraction length of the SFM using a mechanical structure. If electric power is supplied only during the contraction of the SFM and if the contraction is maintained using a mechanical locking mechanism after contraction, the running time of the battery can be improved by reducing the power consumption of the battery. To this end, it is necessary to conduct future studies on locking/unlocking mechanisms that can mechanically select the contracted/relaxed state of the SFM. The prototype STWR proposed in this paper uses a method in which a wearer turns the BOA manually to fix it on the body. However, in future, more advanced STWRs could be developed if the turning of the BOA can be automated and the STWR can recognize the intention of wearers and determine automatically whether assistive force should be provided. Also, to improve the cyclic response of STWR motion, it is necessary to fabricate SMA springs inside SFM with smaller diameter wire or to study additional active cooling method. In future, a study will be carried out to develop an STWR that will facilitate force and position controls according to the intention on various body parts by including a motion recognition function in the STWR.

## Methods

### Position control of SFM

The SFM was fabricated using methods described by Park *et al*.^31^. The K-type thermocouple (TT-K-30-SLE, Omega Engineering Inc., Republic of Korea) was attached to one of the shape-memory-alloy springs to monitor the temperature of the SFM by using Kapton tape (S-7595, Uline, WI, USA). The position controller was implemented using MATLAB/SIMULINK xPC Target, and the analog voltage output of the wire encoder was measured by an NI DAQ board (PCI-6221, National Instruments Corp., TX, USA). The position controller outputs current commands to the current driver (JSP-180-30, Copley Controls, MA, USA) to control the temperature and contraction length of the SFM.

### Statement of consent

The two persons in ‘Supplementary video S2.mp4’ are the authors ‘Seong Jun Park’ and ‘Cheol Hoon Park’, and the person in ‘Fig. 3c’ is the author ‘Cheol Hoon Park’. We consent for publication of identifying information/images in an online open-access publication.

## Supplementary information

Supplementary Video S1

Supplementary Video S2

## Data Availability

The authors declare all data is available.
